# Relationships between resource availability and elevation vary between metrics creating gradients of nutritional complexity

**DOI:** 10.1007/s00442-020-04824-4

**Published:** 2021-01-18

**Authors:** Mark A. Lee, Grace Burger, Emma R. Green, Pepijn W. Kooij

**Affiliations:** 1 Natural Capital and Plant Health, Royal Botanic Gardens Kew, Richmond, TW9 3AB UK; 2 Comparative Plant and Fungal Biology, Royal Botanic Gardens Kew, Richmond, TW9 3AB UK; 3 School of Natural Sciences, Bangor University, Gwynedd, LL57 2DG UK; 4 Center for the Study of Social Insects, São Paulo State University (UNESP), Rio Claro, SP 13506-900 Brazil

**Keywords:** Altitude, Biodiversity, Forage, Grassland, Protein

## Abstract

Plant and animal community composition changes at higher elevations on mountains. Plant and animal species richness generally declines with elevation, but the shape of the relationship differs between taxa. There are several proposed mechanisms, including the productivity hypotheses; that declines in available plant biomass confers fewer resources to consumers, thus supporting fewer species. We investigated resource availability as we ascended three aspects of Helvellyn mountain, UK, measuring several plant nutritive metrics, plant species richness and biomass. We observed a linear decline in plant species richness as we ascended the mountain but there was a unimodal relationship between plant biomass and elevation. Generally, the highest biomass values at mid-elevations were associated with the lowest nutritive values, except mineral contents which declined with elevation. Intra-specific and inter-specific increases in nutritive values nearer the top and bottom of the mountain indicated that physiological, phenological and compositional mechanisms may have played a role. The shape of the relationship between resource availability and elevation was different depending on the metric. Many consumers actively select or avoid plants based on their nutritive values and the abundances of consumer taxa vary in their relationships with elevation. Consideration of multiple nutritive metrics and of the nutritional requirements of the consumer may provide a greater understanding of changes to plant and animal communities at higher elevations. We propose a novel hypothesis for explaining elevational diversity gradients, which warrants further study; the ‘nutritional complexity hypothesis’, where consumer species coexist due to greater variation in the nutritional chemistry of plants.

**Supplementary Information:**

The online version of this article (10.1007/s00442-020-04824-4) contains supplementary material, which is available to authorized users.

## Introduction

Upland mountain ecosystems are biologically distinct from nearby lowlands. Ecological studies have sought to quantify and explain plant and animal community changes with elevation since the early pioneers such as Alexander von Humboldt and Charles Darwin. Plant and animal species richness, the abundances of individual species or functional groups as well as soil, plant and animal interactions have been correlated with elevation (Craine and Lee 2003; Mori et al. 2013). However, there is a great deal of variation in the magnitude, shape and direction of these relationships as well as in the proposed mechanisms (Grytnes and McCain 2013).

Linear, quadratic (plateau-shaped) and unimodal (hump-shaped, peaking at mid-elevations) declines in plant and animal diversity with elevation have been reported (Grytnes and McCain 2013). Unimodal declines in species richness may be the most common pattern for plants but is not the most common pattern for other taxa (Grytnes 2003; McCain 2005, 2007, 2010). There are several proposed mechanisms for understanding the decline in species richness at higher elevations. These are; (1) the ‘temperature hypothesis’ and (2) the ‘water availability hypothesis’ where cooler temperatures or lower water availability reduces the rate and complexity of biotic interactions (e.g. competition, facilitation); (3) the ‘productivity hypothesis’ where reduced plant productivity limits population sizes and therefore species persistence and coexistence; (4) the ‘area hypothesis’, where steeper slopes reduce land area; (5) the ‘geometric constraints hypothesis’, where upland species are constrained by dispersal and (6) the ‘plant diversity hypothesis’, where habitat complexity is reduced by a lower diversity of plants at higher elevations (Peters et al. 2016).

Colder temperatures and drier soils are frequently correlated with reduced plant productivity and reduced species richness at higher elevations and so the temperature-, water availability- and productivity hypotheses are commonly supported in elevational gradient studies (McCain 2007; Kessler et al. 2009). Plant productivity was correlated with species richness in eight plant and animal taxa out of a total of 25 on Mt Kilimanjaro; however, positive and negative effects of primary productivity were equally common (Peters et al. 2016). A key assumption of the productivity hypothesis is that greater plant productivity is of enhanced value to herbivores and can support more herbivore species. However, there is substantial variation in the nutritional value of plants; both within- and between plant species. Plants can vary in terms of protein (2–36%), fibre (23–90%), lignin (1–21%) and mineral contents (2–22%), as well as in their water contents (3–89%), vastly changing their value to consumers (Lee 2018). Growing conditions and phenology can also modify these nutritive values and therefore greater plant biomass or productivity may not represent greater availability of resources (Lee et al. 2014, 2017).

Plant biomass commonly declines with elevation due to the adverse growing conditions (Wang et al. 2007). However, foliar nitrogen (N) can be enhanced at high elevation in some alpine species, and high foliar N is associated with greater nutritional value to some herbivores (Körner 1989; Cordell et al. 1999). Similarly, unpalatable defensive leaf compounds (e.g. tannins and silica) can decrease at higher elevations in some plant species (Alonso-Amelot et al. 2004). Although there have been some studies which have investigated elevational patterns in foliar N or in the foliar carbon:nitrogen (C:N) ratio, there are many metrics of nutritional value and these can be more informative for animal health and fecundity (Cameron et al. 2018). Plant protein and mineral contents are generally positively associated with nutritive value to herbivores whereas fibre is generally associated with lower nutritive value, depending on the herbivore species (Waghorn and Clark 2004). To our knowledge, an assessment of changes to different plant nutritive metrics with elevation is currently lacking, limiting our ability to disentangle the proposed elevational diversity hypotheses.

We sought to quantify variation in plant species richness and nutritive values for herbivores, and to understand the underlying drivers of change, along an elevational gradient. Our approach incorporated multiple metrics of plant nutritive values along three different aspects of an iconic mountain, Helvellyn, situated in the Lake District National Park, north-west England. We measured variation in plant community composition (species and functional groups), plant nutritive values to herbivores (protein, fibre, minerals and C:N) and plant biomass as we ascended the mountain. We also recorded slope, soil moisture and soil temperature as potentially confounding variables. We hypothesised; (1) that plant species richness, plant biomass and plant nutritive values would follow unimodal relationships with elevation and (2) that increased plant biomass at mid-elevations would be associated with greater nutritive value to herbivores.

## Materials and methods

### Site

**Fig. 1:**
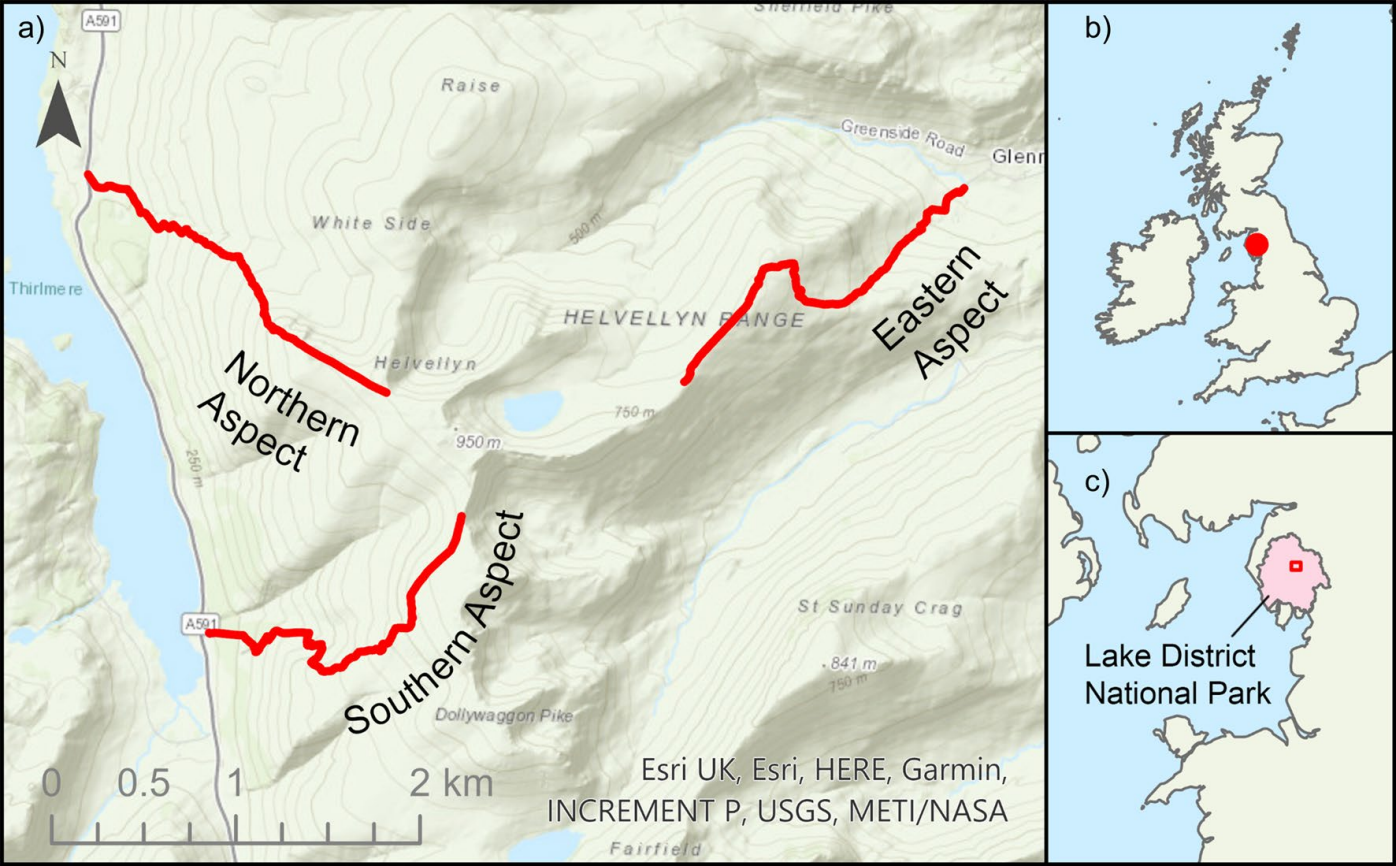
**a** Topographic map of Helvellyn detailing the three sampling routes along the three aspects denoted in red. **b** The location of the Lake District National Park within the United Kingdom. **c** The location of Helvellyn (red square) within the Lake District National Park

The Lake District is situated in the north-west of England (Fig. 1), and covers an area of over 2,000 km^2^, making it the largest National Park in England. The Lake District National Park was designated a UNESCO world heritage site in 2017. The Lake District is made up of an intricate topography due to previous heavy glaciation forming approximately 150 peaks and many surrounding lakes and tarns. The geology of the Lake District is comprised of Borrowdale volcanic rock, Ordovician slates and Silurian shale. Its diverse landform and geology support a variety of upland habitats, including grassland, heathland, woodland, scrub, mires, freshwater lakes and alpine/montane regions above 700 m. The climate of the Lake District is relatively mild and wet (mean maximum temperature = 13 °C, mean minimum temperature = 6 °C, mean annual rainfall = 2005 mm, 1981–2010) due to its proximity to the Irish Sea and the Atlantic Ocean to the west (Met Office 2017).

Our focal mountain, Helvellyn, is situated centrally within the Lake District National Park and is the third highest peak in England at approximately 950 m high (54.5268° N, 3.0172° W). The mountain can be characterised by its steep glacial valleys and impoverished high-altitude acidic grassland communities. The diverse topology of Helvellyn supports an equally diverse collection of habitats, to include stands of *Juniperus communis* on upland acidic soil, higher altitude areas of siliceous alpine grasslands, species-rich *Nardus* grassland and rocky cliff ledges and scree habitats supporting rare montane plant species (JNCC 2015). The grazing density is kept to one ewe per hectare in the summer and reduced to approximately one ewe per two hectares in the winter (S. Webb, *pers. comms*, 2018). Grazers were allowed to roam freely within our study area.

### Sampling design

Three aspects of Helvellyn were surveyed and sampled over the course of two weeks in May and June 2018, covering the SW (hereafter termed ‘southern’), NW (hereafter termed ‘northern’) and NE (hereafter termed ‘eastern’) aspects. The SE aspect was inaccessible for sampling and was therefore excluded. The surveys focused on upland grassland vegetation, sampling from 300 m to 850 m above sea level (asl) on the southern and northern aspects and 300 to 700 m asl on the eastern aspect. Surveying at 850 m asl on the eastern aspect was not possible. Surveys were carried out at 100 m elevation intervals from 300 m asl; excluding 800 m asl due to a band of scree, and instead surveying at 850 m asl where there was suitable vegetation. Locations above 900 m asl were not suitable for vegetation sampling due to scree.

At each elevation and for all three aspects, five 25 cm^2^ (50 cm × 50 cm) quadrats were randomly positioned along 25 m transects using a random number generator that began 1 m from the path edge and ran perpendicular to the path at a constant elevation. The random number represented the number of metres from the main path to where the quadrats would be placed. Within each quadrat, plant cover was visually estimated for all species. Slope was visually estimated for each quadrat, and soil temperature and soil moisture were measured at four randomly allocated locations using a digital handheld probe at 5 cm soil depth (Omega; Manchester, UK). We measured edaphic conditions four times for each of the five samples at every elevation and across all three transects (total samples = 360). Soil temperature and moisture were used as these values are more representative of prevailing climate than air temperature or rainfall, which changes throughout the day. Vegetation height was also measured at five randomly allocated locations within each quadrat. Plant functional group composition and growth forms were also recorded. Classification of plant growth forms was based on a standardised classification of plant functional traits (Pérez-Harguindeguy et al. 2013). We assigned plant growth forms based on the amount and direction of growth, and the degree of branching of the main-shoot, categorising each species as short basal, long basal, semi-basal, erect leafy, cushions, tussocks or dwarf shrubs. Growth form is indicative of adaptative traits, such as those which maximise photosynthesis, increase tolerance of extreme growing conditions, or reduce the ease of access or palatability of foliage for grazing herbivores.

Three separate randomly allocated quadrats on the northern and southern aspects were cut to ground level at each elevation. These samples were weighed (hereafter termed ‘fresh biomass’) and then dried at 60 °C for 48 h and re-weighed (hereafter termed ‘dry biomass’). Subtracting dry biomass from fresh biomass provided plant water contents. These samples were then used for plant community assessments of nutritive values; crude protein (CP), mineral (ash) contents and neutral detergent fibre (NDF) contents. Additional samples of the three dominant grass species; *Anthoxanthum odoratum*, *Festuca ovina* and *Nardus stricta* were also collected at each elevation, sampled randomly from healthy individuals on the northern and southern aspects. Six samples from each grass were harvested at every sampling point. These samples were air dried and stored in paper bags for carbon:nitrogen ratio (C:N) analysis.

### Analyses of plant nutritive values

Plant carbon (C), nitrogen (N) and C:N were measured using mass spectrometry and the Dumas dry combustion method. This method involves total combustion of samples and reduction of the gases using copper. Carbon and nitrogen are then measured using a universal detector (Marcó et al. 2002). Samples of the three grass species were dried and finely ground using a TissueLyser LT (Qiagen, Venlo, Netherlands), selecting the younger leaf blades for sampling consistency. The ground samples were then weighed in a tin capsule on a balance to obtain a sample weighing between 0.5 and 1 mg, which was then placed into the C:N autosampler. Recalibration of the mass spectrometer was carried out after every 10 samples, placing one empty tin capsule (blank) and two capsules containing casein into the autosampler alongside the samples.

Paired samples were ground to a fine powder and 1 g sub-samples were used for each nutritional analysis. Mineral ash values represented the inorganic mineral component of the plants and were measured by burning samples at 500 °C for two hours and weighing the residual component. Neutral detergent fibre (NDF) represented the fibre content of forage comprising lignin, silica, cellulose and hemicellulose. NDF was measured using modified neutral detergent analysis (Van Soest et al. 1991). Forage protein content was included as crude protein and was measured by Kjeldahl digestion using sulphuric acid and analysed by steam distillation using a Gerhardt–Vadopest system (Gerhardt Vadopest 6, Germany). The dry matter (DM) content of each sample was also included as the proportion of material remaining following drying (60 °C for 48 h), as well as the plant water content (100-DM). All methods are standard wet chemistry forage analyses (AOAC 2000).

### Statistics

Linear and nonlinear relationships (quadratic and logarithmic) were fitted between elevation and abiotic variables (soil temperature and moisture) and biotic variables (species richness, Shannon diversity, biomass, height, functional groups and nutritive values). The Shannon diversity metric was used to represent both species richness and abundance at each sampling locations. Relationships between variables were tested for significance using linear and nonlinear regression with the optimal relationship identified using analysis of variance (ANOVA) tests. More complex, nonlinear, relationships were only selected if they were a significantly better fit to the data, otherwise the simpler linear relationships were chosen (Crawley 2013). Data were checked for normality prior to statistical testing.

The multidimensional dataset was further analysed using principal component analysis (PCA) to visualise the patterns of association. Due to missing data for the eastern aspect for some of the variables (plant biomass and nutritive values), only the southern and northern aspects were included in the analysis. Data were analysed using the FactoMineR and FactoExtra packages and visualised with the fviz_pca_var and fviz_pca_ind functions. Aspect and elevation were included as categorical variables followed by the slope, soil temperature, soil moisture, mineral ash, CP and NDF as quantitative variables and the grass-, sedge-, rush-, fern-, herb-, legume-, moss-abundance, species richness, bare ground, vegetation height and the Shannon index as predictor variables.

## Results

### Plant species richness

**Fig. 2:**
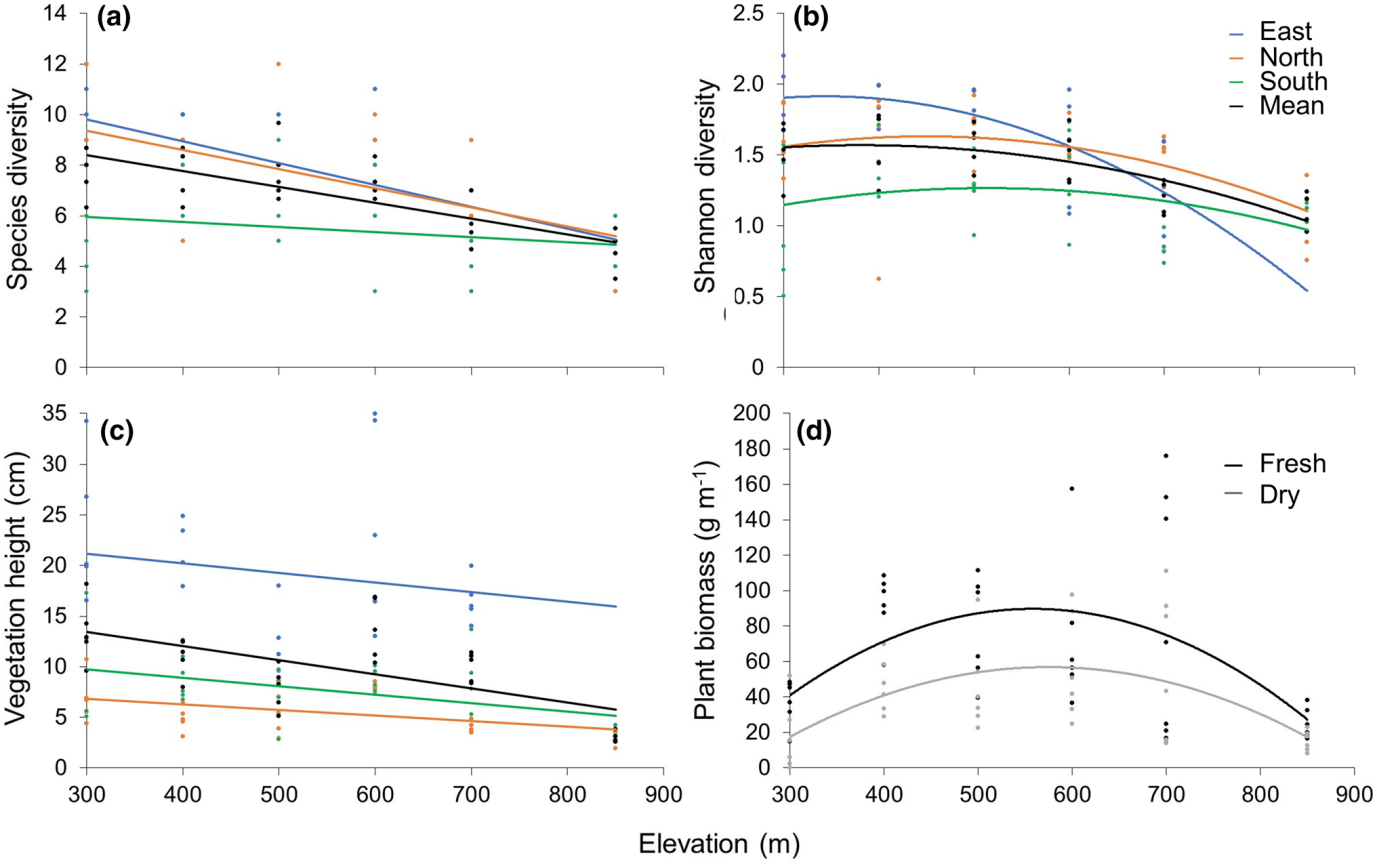
Relationship between elevation and **a** species richness (Richness = − 9.8*x* + 6.91, *F* = 24.8, *r*^2^ = 0.23, *P* < 0.001), **b** Shannon diversity (Shannon diversity = − 0.71*x*^2^ − 1.46*x* + 1.43, *F* = 10.5, *r*^2^ = 0.20, *P* < 0.001), **c** vegetation height (Height = − 19.8*x* + 10.3, *F* = 7.4, *r*^2^ = 0.08, *P* < 0.01) and **d** dry biomass (Dry biomass = − 0.0005*x*^2^ + 0.6*x* − 115, *F* = 8.4, *r*^2^ = 0.32, *P* < 0.01) and fresh biomass (Fresh biomass = − 0.0007*x*^2^ + 0.8*x* − 138, *F* = 6.8, *r*^2^ = 0.28, *P* < 0.01). Biomass values represent means of all aspects

Plant species richness declined with elevation from a mean of 8 species at 300 m asl to a mean of 5 species at 850 m asl (Fig. 2a). Species richness also varied between aspects, with the eastern and northern aspects being generally more diverse than the southern aspect (*F* = 14.3, *P* < 0.001). This difference was more marked nearer the bottom of the mountain. At 300 m asl, the southern aspect had a mean of 5 fewer species than both the northern and eastern aspects, whereas by 700 m asl this difference had declined to 2 species and by 850 m asl there was no difference between aspects. There was a concurrent decline in the Shannon diversity index with elevation, but this followed a nonlinear pattern (Fig. 2b). There were also differences in the Shannon diversity index between aspects (*F* = 15.7, *P* < 0.001), with the eastern and northern aspects generally having higher values than the southern aspect.

The total number of unique species identified across all aspects and quadrats also declined with elevation, from 16 species being recorded at 300 m asl to 6 species being recorded at 850 m asl. Some species were only found at 300 m asl, for example, *Cardamine flexuosa*, *Digitalis purpurea*, *Eriophorum angustifolium* and *Oxalis acetosella*. The low palatability grass, *N. stricta*, and the mosses, *Polytrichum* spp, were most abundant at mid-elevations (500 m-700 m asl) whilst other species showed a patchy distribution. *F. ovina* increased in abundance with elevation and was the dominant species at 850 m asl. Only five other species were recorded in our quadrats at 850 m asl; *A. odoratum*, *Campylopus introflexus*, *Galium saxatile*, *Vaccinium myrtillus* and *Polytrichum* spp (for a list of species see the ESM). Bare ground followed a nonlinear relationship with elevation, increasing nearer the top and bottom (Table 1).

**Table 1:** Relationships between the stated variable and elevation, as identified by linear and nonlinear regression, in the format: *ax*^2^ + *bx* + intercept (*ax*^2^ only presented if the nonlinear model was optimal)

Variable	*bx*	*ax*^2^	Intercept	*R*^2^	*F*	DF	*P*
Grass	84.04	− 59.71	61.95	0.19	9.63	82	< 0.001
Rush	− 28.86		4.19	0.07	6.16	83	< 0.05
Sedge	− 0.02		19.93	0.11	9.97	83	< 0.01
Fern	− 1.47		0.19	0.04	3.39	83	0.07
Non-legume herb	− 15.48		11.72	0.03	2.36	83	0.13
Legume herb	− 0.74		0.34	0.00	0.27	83	0.60
Moss	13.97	− 25.56	12.45	0.07	2.94	83	0.06
Bare ground	64.57	− 20.29	6.67	0.24	13.13	82	< 0.001
Short basal	− 8.67	6.48	0.89	0.17	8.41	82	< 0.001
Long basal	− 1.47		0.19	0.04	3.39	83	0.07
Semi-basal	− 12.64		7.12	0.05	4.05	83	< 0.05
Erect leafy	− 9.40		1.46	0.09	7.94	83	< 0.01
Cushions	13.97	− 25.56	12.45	0.07	2.94	83	0.06
Tussocks	18.59	− 52.28	73.73	0.08	3.65	82	< 0.05
Dwarf shrubs	14.50		2.59	0.08	6.05	83	< 0.05

### Plant functional diversity

The mean coverage of grasses increased with elevation, but the relationship was nonlinear (Table 1). The most common grass species recorded were *A. odoratum*, *F. ovina* and *N. stricta*. At the lowest elevation grass coverage was a mean of 39% increasing to a peak of 80% coverage at 700 m asl. As coverage of grasses increased the coverage of rushes and sedges declined, with these groups declining from a mean coverage of 9% and 13%, respectively, at 300 m asl, to not being present at 850 m asl. The sedges that were recorded were *Carex bigelowii*, *Carex canescens*, *Carex flava* and *Carex hirta*, whilst *Juncus effusus* was the only rush that was recorded. The abundances of the remaining plant families were not related to elevation, although the fern, *Pteridium aquilinum*, was only recorded at lower elevations, between 300 m asl and 500 m asl.

The coverage of semi-basal and erect leafy plants declined linearly with elevation, however, the abundance of dwarf shrubs increased. Tussocks and short basal plants were nonlinearly related to elevation. Tussocks were lowest in abundance at the top and bottom elevations, peaking at mid-elevations. Conversely, short basal plants increased in abundance at 300 m asl and 400 m asl but were not present at 500, 600 or 700 m asl.

### Plant biomass and nutritional chemistry

Mean vegetation height declined with elevation from a mean of 14 cm at the lowest sampling point to a mean of 3 cm at the highest (Fig. 2c). There was also variation between aspects (*F* = 67.0, *P* < 0.001), with vegetation height of the eastern aspect a mean of 13 cm taller than the other two aspects. Both fresh and dry biomass followed nonlinear, unimodal relationships with elevation and the lowest biomass values were recorded at the lowest and highest elevations, peaking at the mid-point; 500–600 m asl (Fig. 2d).

**Fig. 3:**
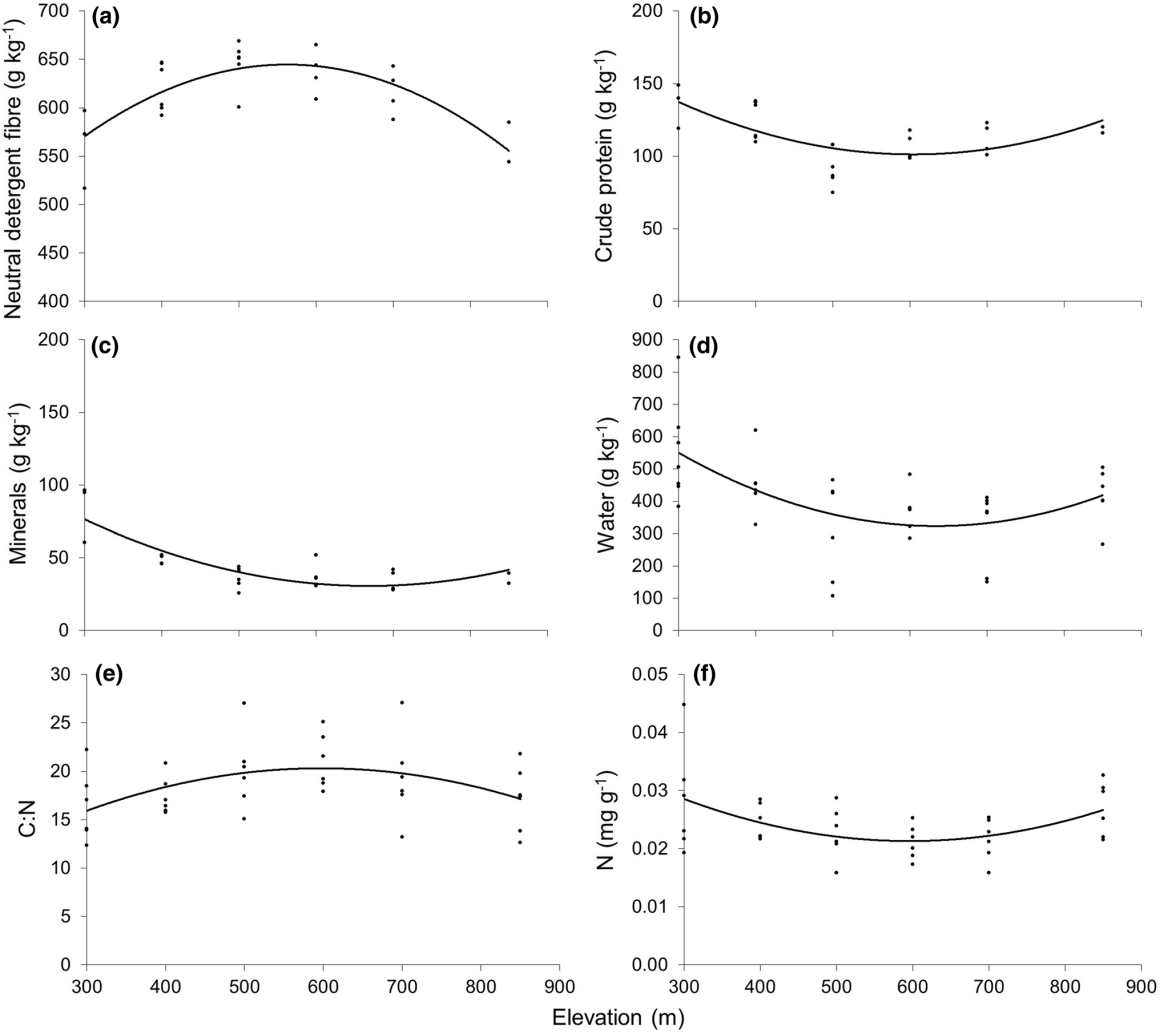
Nonlinear relationships between community plant **a** neutral detergent fibre (NDF = − 142.1*x*^2^ + 1.2*x* + 617.4, *F* = 15.2, *r*^2^ = 0.24, *P* < 0.001), **b** crude protein (CP = 51.5*x*^2^ − 26.1*x* + 112.9, *F* = 8.0, *r*^2^ = 0.22, *P* < 0.01), **c** mineral ash (minerals = 44.6*x*^2^ − 56.0*x* + 45.4, *F* = 25.4, *r*^2^ = 0.70, *P* < 0.001) and **d** plant water content (water = − 17.3*x*^2^ − 6.0 + 6.5, *F* = 3.1, *r*^2^ = 0.1, *P* = 0.06). Nonlinear relationships between the dominant three grass species and **e** carbon:nitrogen ratio (C:N = − 9.2*x*^2^ + 2.5*x* + 18.6, *F* = 3.9, *r*^2^ = 0.19, *P* < 0.05) and **f** nitrogen contents (N = 0.02*x*^2^ + 0.001*x* + 0.02, *F* = 5.13, *r*^2^ = 0.24, *P* < 0.05)

All the nutritional chemistry measurements were nonlinearly related to elevation, except carbon contents which were not related (*P* > 0.05, Fig. 3). Low palatability metrics, community mean NDF and C:N of the dominant three grass species, showed a similar unimodal relationship with elevation as biomass, with mean values lowest at the lowest elevation and highest elevation, peaking at the mid-points; 500–600 m asl. The C:N ratio differed between plant species, with the mean C:N being 6.1 and 2.7 times greater for *F. ovina* and *N. stricta*, than for *A. odoratum*, respectively. High palatability metrics, community CP, plant water contents and N contents of the dominant three grasses followed an approximately inverse, unimodal relationship to NDF and C:N, with mean values peaking at the highest and lowest elevations and declining at mid-elevations. Although the relationship between plant water contents and elevation was not significant (*P* = 0.06). The only deviation from the unimodal patterns was plant mineral contents which peaked at the lowest elevation, declining to a baseline level from 500 m asl.

**Fig. 4:**
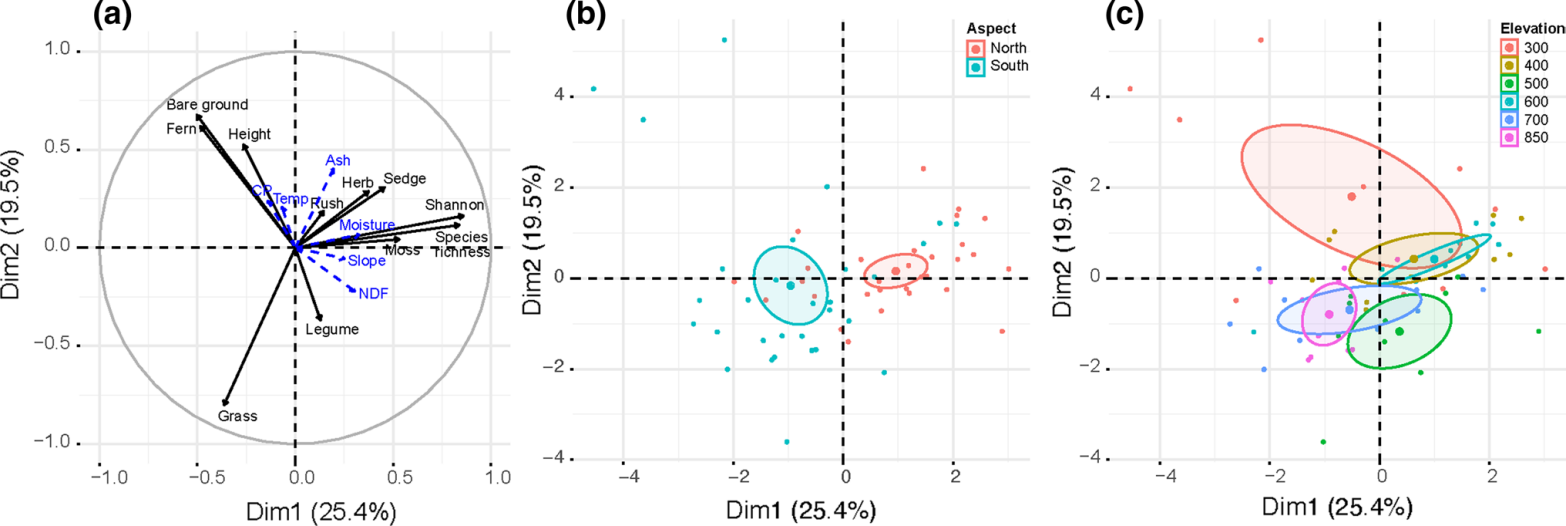
PCA using biotic parameters as predictor variables in which **a** predictor variables are shown using black arrows and the quantifying variables using blue arrows, and individual plots are coloured using either **b** aspect or **c** elevation as a category. The outlines represent 95% confidence intervals

PCA revealed that the first, second and third dimension of the PCA had, as the most important predictor variables; the Shannon index (*R*^2^ = 0.86, *P* < 0.001), grass cover (*R*^2^ = −0.80, *P* < 0.001, and rush cover (*R*^2^ = −0.67, *P* < 0.001), respectively (Fig. 4a). In general, greater plant species richness was associated with increases in the abundances of herbs, rushes, and sedges, as well as with higher soil moisture. Increased fern abundance and bare ground were associated with an increase in nutritive values in terms of plant CP contents, alongside an increase in soil temperatures. Increased abundances of grasses and herbaceous legumes were associated with increases in NDF contents and a decline in nutritive values. Species richness was unrelated to plant biomass (*P* > 0.05), but species richness did increase linearly with slope (*t* = 2.7, *P* < 0.01). Overall, PCA revealed clear differences in the biotic variables between the northern and southern aspects (*R*^2^ = 0.33, *P* < 0.001, Fig. 4b) and across the different sampling elevations (*R*^2^ = 0.47, *P* < 0.001, Fig. 4c).

## Discussion

We observed a decline in plant species richness and in the Shannon diversity index as we ascended the mountain of Helvellyn, across all three aspects. This was driven in large part by a reduction in the abundances of the majority of plant species alongside an increase in six species at high elevations, which was dominated by the grass, *F. ovina*. This finding is in line with several other studies reporting declines in plant species richness at high elevations (Grytnes and McCain 2013; Sundqvist et al. 2013). Community plant biomass (both dry and fresh biomass) did not follow the same pattern as plant species richness instead following a unimodal relationship, peaking at mid-elevations, and declining at high and low elevations. The increase in biomass at mid-elevations was associated with increases in the abundance of plants in the tussock functional group with several of the other functional groups declining.

Greater plant biomass has been associated with greater consumer species richness, population persistence, increased population sizes, and fewer extinctions (Hanski and Thomas 1994; Schlinkert et al. 2016). However, the quality and palatability of plants also affect the amount of vegetation that is consumed, rates of animal weight gains and reproductive success (Herrero et al. 2013). Low nutritive value diets can lead to higher mortality rates, lower pregnancy rates, production of fewer offspring and a higher risk of predation (Proffitt et al. 2016). In our study, the highest biomass values at mid-elevations were generally associated with the lowest plant community nutritive values (high fibre, low protein and low water contents). This finding is in line with other studies which have not found consistent relationships between plant biomass and species richness across many taxa (Peters et al. 2016) and is also in line with studies which have not found unimodal relationships between species richness and elevation—decreasing, low-elevation plateau and low-elevation plateau with a mid-peak patterns have been reported for non-flying small mammals (McCain 2005), bats (McCain 2007), birds (McCain 2009), reptiles (McCain 2010) and plants (Rahbek 2005).

The three dominant grasses also displayed lower nutritive values at mid-elevations, as well as the community level measurements. These results suggest that both intra-specific changes within the dominant three species as well as inter-specific compositional changes were both responsible for the reductions in nutritive values that were recorded. Reduced nutritive values within species may have been driven by phenological and physiological changes such as advanced flowering dates, modified stem:leaf ratios, thicker cell walls and increased lignification, which may be caused by variation in abiotic conditions (Hirata et al. 2008; Kering et al. 2011; Gardarin et al. 2014; Lee et al. 2019). Plant compositional changes were also associated with reduced nutritive values, likely due to increases in the abundance of unpalatable tussock plants, such as *N. stricta* (Parolo et al. 2011). PCA supported this assertion due to the positive association between fibre content and grass cover.

Most of the metrics of palatability indicated that plants were generally of greater nutritive value nearer the summit and base of the mountain, but plant mineral content showed a different pattern to the other nutritive metrics and declined with elevation, alongside plant height and species richness. Increased plant height at low elevations was associated with low biomass. These patterns may have been driven by greater abundances of semi-basal and erect leafy plants, with the herbaceous plants, sedges and rushes most common at low elevations. Arthropod diversity frequently declines with elevation and this may be due to a decline in plant nutritive values and/or habitat complexity, however, declines in the abundance of specific host plants will also reduce the abundance of specialist feeding arthropods (Hodkinson 2005). Future studies investigating elevational gradients should consider the nutritional requirements of consumers as well as the nutritional chemistry of plants.

Complex patterns of plant nutritional value changes with elevation could influence the abundance and diversity of other taxa. Birds, mammals, amphibians, reptiles and arthropods actively select or avoid plants based on their nutritive values (Greenberg and Bichier 2005; Amato and Garber 2014). Such variation may contribute to niche segregation, to the coexistence of herbivore populations and to increased species richness (Redjadj et al. 2014; Kartzinel et al. 2015). Animal behaviour can also be influenced by plant nutritional values and it has been shown that wildebeest and zebra travel greater distances and remain within grazing patches for shorter periods when forage is of high nutritive value (Hopcraft et al. 2014). In our study, PCA revealed that the Shannon index was the strongest predictor variable for variation in the plant nutritive metrics. This indicates that species richness and evenness may contribute towards shaping patterns of nutrition and thus resource availability on mountains. We therefore propose a novel hypothesis for explaining elevational diversity gradients, which requires rigorous scientific testing; the ‘nutritional complexity hypothesis’ in which consumer species coexist due to greater variation in the nutritional chemistry of plants caused by physiological, phenological and compositional changes. Such changes can be modified by factors such as slope, temperature and water availability (Lee et al. 2010) which have been associated with elevational diversity patterns (McCain 2007).

Higher elevation plants may be exposed to less competition for nitrogen or other nutrients than plants of the same species at lower elevations growing at higher densities (Körner 1989). It may also be the case that grazing pressure may be lower, leaving more palatable individuals (Graff et al. 2007). Increased photosynthetic efficiency, increased leaf N and reduced unpalatable defensive compounds have been observed where herbivory pressure declines and with increasing elevation (Hodkinson 2005; Dostálek et al. 2016). Although sheep do graze this site, we do not know whether there is any variability in grazing pressure with elevation. Further work is needed to disentangle the mechanisms for the variability in plant nutritive values with elevation, although we provide tentative evidence that soil temperature, soil moisture and/or a covariate may play a role. Nevertheless, we present evidence of a mismatch between plant biomass and nutritive values and of different relationships between the different metrics of resource availability and elevation. Greater consideration of the nutritional requirements of different consumer species and quantification of how nutrition changes in space and time may increase our understanding of elevational diversity gradients.

### Limitations

Seasonal variation in elevational gradients including in plant nutritive values and herbivory have been observed and were outside the scope of this study (Suzuki 1998). We included spot measurements of soil temperatures and soil moisture as covariates in our PCA, which we could not take simultaneously across all sampling locations. Soil conditions can change over days, months and years, modifying the patterns we observed. However, we measured soil temperature and moisture because these values show much less variability over shorter time scales than air temperature and rainfall. Soil temperature and soil moisture were included to acknowledge their potential impact on the patterns we observed but were not the focus of this study. We did not measure variability in soil fungi, which can vary with elevation (Kotilínek et al. 2017). Soil organic carbon and microbial biomass can also be modified by elevation and other edaphic conditions may have contributed to the patterns we observed (Tan and Wang 2016). Alternatively, spatial variation in plant and herbivore community composition could be regulated by processes that operate at larger scales (e.g., speciation, extinction and dispersal) which may have also influenced our results (Hanski and Thomas 1994). However, there is a great deal of evidence that bottom-up processes play a large role in determining plant and herbivore population sizes and species richness along elevation gradients (Sundqvist et al. 2013).

## Conclusions

Plant and animal community composition changes at higher elevations and this is generally associated with a decline in species richness. There are several hypotheses for understanding these relationships, but none have gathered sufficient evidence to build a consensus. This may be because many herbivores actively select or avoid plants based on their nutritive values, but variation in nutrition has not previously been included. We adopted a novel approach for understanding elevational diversity gradients by quantifying changes to several metrics of resource availability. Greater plant biomass at mid-elevations was associated with lower plant nutritive values, indicating that greater plant productivity may not confer greater resources to herbivores. This is not consistent with the ‘productivity hypothesis’ which assumes that greater biomass leads to greater resource availability. Furthermore, the shapes of the relationships between plant nutritive values and elevation changed depending on the metric. Individual metrics of resource availability (such as biomass, C:N or fibre contents) are insufficient to fully describe variation in plant nutritional value to consumers. The Shannon diversity index best explained variation across the nutritive value metrics and plant species diversity and evenness may contribute towards increased nutritional complexity. Consideration of the nutritional requirements of herbivores at different times in their lifecycles and quantification of the nutritive values of plants, using different nutritional metrics, may contribute to an increased understanding of elevational diversity gradients. We propose a novel hypothesis for understanding biodiversity gradients which warrants further study; the ‘nutritional complexity hypothesis’ in which consumer species coexist due to greater variation in the nutritional chemistry of plants over space and time caused by physiological, phenological and compositional changes driven by elevation.

## Supplementary Information

Below is the link to the electronic supplementary material.

Supplementary material 1 (DOCX 15 kb)
