# Supplementary material for: Relationships between resource availability and elevation vary between metrics creating gradients of nutritional complexity
Source: Oecologia. 2021 Jan 18;195(1):213–23. doi: 10.1007/s00442-020-04824-4 (PMC7882561; doi:10.1007/s00442-020-04824-4)
Supplement: Supplementary file 1 — Supplementary material 1 (DOCX 15 kb) [file 442_2020_4824_MOESM1_ESM.docx]

Electronic supplement to manuscript

**Relationships between resource availability and elevation vary between metrics creating gradients of nutritional complexity**

**Mark A. Lee^1^*, Grace Burger^1,2^, Emma R. Green^3^ and Pepijn W. Kooij^2,4^**

^1^ Natural Capital and Plant Health, Royal Botanic Gardens Kew, Richmond, TW9 3AB, UK

^2^ Comparative Plant and Fungal Biology, Royal Botanic Gardens Kew, Richmond, TW9 3AB, UK

^3^ School of Natural Sciences, Bangor University, Gwynedd, LL57 2DG, UK

^4^ Center for the Study of Social Insects, São Paulo State University (UNESP), Rio Claro - SP, 13506-900, Brazil

*Corresponding author: E: Mark A. Lee (m.lee@kew.org)

Table ESM 1: Species list across all sampling points.

| Species |
| --- |
| Achillea millefolium |
| Anthoxanthum odoratum |
| Avenella flexuosa |
| Brachythecium albicans |
| Campylopus introflexus |
| Cardamine flexuosa |
| Carex bigelowii |
| Carex canescens |
| Carex flava |
| Carex hirta |
| Cirsium palustre |
| Convolvulus arvensis |
| Digitalis purpurea |
| Drosera rotundifolia |
| Eriophorum angustifolium |
| Festuca ovina |
| Galium saxatile |
| Juncus effusus |
| Nardus stricta |
| Narthecium ossifragum |
| Oxalis acetosella |
| Pedicularis sylvatica |
| Pinguicula vulgaris |
| Plantago lanceolata |
| Poa alpina |
| Polygala serpyllifolia |
| Polytrichum spp. |
| Potentilla erecta |
| Pteridium aquilinum |
| Ranunculus repens |
| Rumex acetosa |
| Senecio sylvaticus |
| Sphagnum spp. |
| Stellaria media |
| Trifolium repens |
| Vaccinium myrtillus |
| Veronica chamaedrys |
